# SDF-1 Enhances Wound Healing of Critical-Sized Calvarial Defects beyond Self-Repair Capacity

**DOI:** 10.1371/journal.pone.0097035

**Published:** 2014-05-06

**Authors:** Qiming Jin, William V. Giannobile

**Affiliations:** 1 Department of Cariology, Restorative Sciences and Endodontics, School of Dentistry, University of Michigan, Ann Arbor, Michigan, United States of America; 2 Department of Periodontics and Oral Medicine, School of Dentistry, University of Michigan, Ann Arbor, Michigan, United States of America; 3 Department of Biomedical Engineering, College of Engineering, University of Michigan, Ann Arbor, Michigan, United States of America; Texas A&M University Baylor College of Dentistry, United States of America

## Abstract

Host blood circulating stem cells are an important cell source that participates in the repair of damaged tissues. The clinical challenge is how to improve the recruitment of circulating stem cells into the local wound area and enhance tissue regeneration. Stromal-derived factor-1 (SDF-1) has been shown to be a potent chemoattractant of blood circulating stem cells into the local wound microenvironment. In order to investigate effects of SDF-1 on bone development and the repair of a large bone defect beyond host self-repair capacity, the BMP-induced subcutaneous ectopic bone formation and calvarial critical-sized defect murine models were used in this preclinical study. A dose escalation of SDF-1 were loaded into collagen scaffolds containing BMP, VEGF, or PDGF, and implanted into subcutaneous sites at mouse dorsa or calvarial critical-sized bone defects for 2 and 4 weeks. The harvested biopsies were examined by microCT and histology. The results demonstrated that while SDF-1 had no effect in the ectopic bone model in promoting de novo osteogenesis, however, in the orthotopic bone model of the critical-sized defects, SDF-1 enhanced calvarial critical-sized bone defect healing similar to VEGF, and PDGF. These results suggest that SDF-1 plays a role in the repair of large critical-sized defect where more cells are needed while not impacting *de novo* bone formation, which may be associated with the functions of SDF-1 on circulating stem cell recruitment and angiogenesis.

## Introduction

Cells are critical to the success of tissue engineering. Any tissue repair and regeneration depends on its tissue-specific differentiated cells to produce its own extracellular matrix. To date, there have been two types of cells used for cell therapy: differentiated/mature cells and undifferentiated/stem cells. Due to limited availability of mature cells, more attention was focused on stem cell therapy. However, *ex vivo* stem cell therapy encounters major challenges on excessive costs for *in vitro* cell manipulation [1], [2], risks for contaminations, pathogen transmission [3], and tumorigenesis [2], [4]. To overcome these shortcomings, application of chemokines to promote “homing” of host circulating stem cells to a wound may provide a potential to increase the number of local stem cells and improve tissue regeneration.

SDF-1 belongs to the C-X-C chemokine family, and exerts multiple biological functions through its receptor CXCR4, a G-protein coupled transmembrane glycoprotein [5], [6]. SDF-1 is produced by various tissue cells such as nerve, skin, muscle, heart, osteoblast, and perivascular cells. SDF-1 not only participates in embryonic development of bone marrow, heart, blood vessel, and brain [7]–[9], but also mediates postnatal blood cell homeostasis [10], [11]. The SDF-1/CXCR4 axis is an important mechanism for homing circulating stem cells into local normal tissues and injured tissues [12], [13]. SDF-1 has been shown to enhance the recruitment of intravenously infused extragenous stem cells into heart and brain ischemic tissues [14], [15]. In diabetic mice, epithelial cells and myofibroblasts are impaired in the production of SDF-1 in the cutaneous wound tissues. After local administration of SDF-1 at the wound site, more epithelial progenitor cells were chemoattracted to the wound area and wound healing were improved [16]. Furthermore, circulating mesenchymal progenitor cells demonstrated participation in BMP-induced ectopic bone formation in mice, and when the CXCR4 antibody was administrated intravenously, the number of circulating mesenchymal stem cells involved in bone formation was decreased. Thus, osteogenesis was impaired. As a chemokine that enhances circulating stem cell migration into *in situ* bone fracture sites and improves bone healing, SDF-1 plays an important role in recruitment of circulating stem cells.

In order to enhance homing of circulating mesenchymal cells into the wound site, wound vasculature needs to be reestablished quickly following injury. Much evidence has shown that platelet-derived growth factor (PDGF) and vascular epithelial growth factor (VEGF) are two important factors to effectively accelerate angiogenesis.

PDGF is a multifunctional growth factor that participates in embryonic development of organs such as kidney, heart, and vasculature [17], [18], and also plays important function in postnatal tissue repair, regeneration and disease development [19], [20]. More importantly, PDGF is a key member to mediate migration and proliferation of pericytes in vessel walls. PDGF-B^−/−^ and PDGFR-beta^−/−^ knock-out mice demonstrated dilated large vessels and deficiency in capillary wall formation [21]. We have previously shown that more blood vessel formation was seen in periodontal osseous defects treated with PDGF-B gene therapy group compared to vector controls [19].

VEGF is a heparin-binding glycoprotein, and regulates endothelial cell proliferation, migration, survival, blood vessel vasodilation and permeability [22], [23]. In addition, VEGF is indispensable to the transition from hypertrophic cartilage to primary ossification during endochondral bone development [24]. VEGF also enhances postnatal bone regeneration through angiogenesis [25].

Although angiogenesis factors such as VEGF and PDGF and chemoattractants such as SDF-1 may improve circulating stem cell homing to local wound area, these stem cells still require signals to differentiate into mature cells so that they can secrete extracellular matrix to participate in damaged tissue repair or regeneration.

BMPs are members of the TGF-β superfamily that are powerful regulators of cartilage and bone formation during embryonic development and regeneration in postnatal life [26]. So far, BMP has been known as a key signaling molecule to induce mesenchymal stem cells to differentiate into osteoblasts and initiate ectopic bone formation. Evidence has indicated that BMP is able to stimulate bone regeneration in bone fracture and around dental implants [27]–[29]. Therefore, BMP can be used to trigger circulating mesenchymal stem cells entering a local wound site to commit bone-forming cell differentiation, and thus enhance bone regeneration.

In order to investigate the effects of SDF-1 on bone development and the repair of a large bone defect beyond host self-repair capacity, BMP-induced subcutaneous ectopic bone formation and calvarial critical-sized defect murine models were used. In addition, the effects of PDGF and VEGF on SDF-1 impacts on calvarial critical-sized defect healing were also explored. Bone formation and wound healing were analyzed by microCT and histology.

## Materials and Methods

### Reagents and Animals

Recombinant human bone morphogenetic protein 7 (BMP-7), recombinant human vascular epithelial growth factor (VEGF), recombinant human platelet derived growth factor (PDGF), and Recombinant Mouse CXCL12/SDF-1 alpha (SDF-1) were purchased from R&D Systems, Inc. (Minneapolis, MN). EDTA and 10% neutral buffered formalin were purchased from Fisher Scientific (Pittsburg, PA). Non-crosslinking resorbable collagen matrix membrane was gifted by Geistlich Pharma AG (Wolhusen, Switzerland). Freshly obtained bovine metatarsal bones were processed to obtain bone powders, which were sieved selectively between 40 and 700 µm in diameter. After decalcification in diluted HCl at a constant pH of 2.0 at 4°C and defatting with methanol and chloroform (volume ratio 1∶1), the powders were extracted four times with 4 M of guanidine HCl, then washed with distilled water and lyophilized. The inactive demineralized insoluble bone matrix (DIBM) was used as the conventional carrier to explore the activity of BMP to induce bone formation. [30]–[33].

### Ectopic Subcutaneous Implantation

All the animal experimental procedures were approved by the University of Michigan Committee of Use and Care of Animals (Protocol Number: PRO00004553). This study complied with ARRIVE guidelines for preclinical animal studies.

In order to investigate the dose-dependent effects of SDF-1 on BMP-induced ectopic bone formation, eight groups were prepared to test in a mouse subcutaneous ectopic bone formation model. They were grouped as follows:

1. Scaffold only (Bovine demineralized insoluble bone matrix, DIBM).
2. 1.0 µg BMP only.
3. 1.0 µg SDF-1.
4. 1.0 µg BMP +10.0 µg SDF-1.
5. 1.0 µg BMP +3.0 µg SDF-1.
6. 1.0 µg BMP +2.0 µg SDF-1.
7. 1.0 µg BMP +1.0 µg SDF-1.
8. 1.0 µg BMP +0.5 µg SDF-1.

To minimize effects of the scaffolds on BMP induced ectopic bone formation, twenty milligrams of bovine demineralized insoluble bone matrix (DIBM) was used as a carrier matrix. DIBM has been previously demonstrated to be devoid of biological activity and BMP, TGF-beta and other growth factor-like activities [30], [34]. Under isoflurane inhalation anesthesia, two mid-sagittal incisions were made on the dorsa of mice, and two surgical subcutaneous pockets were created on both sides of an incision. Each scaffold implant construct was inserted into a surgical pocket. Each mouse had four samples from four different groups. The incisions were stapled closed. There were a total of 9 implants for each group at each time point. Each of these 9 implants was placed in different mouse. A total of 36 mice were used. After biopsies were harvested at 2 weeks and 4 weeks, they were fixed using 10% neutral buffered formalin for 2 days, and then scanned by microCT. Subsequently, three of 9 biopsies of each group were used for histology.

### Calvarial Critical-sized Defects

Under general anesthesia with 100 mg/kg ketamine and 10 mg/kg xylazine, an incision was made along the sagittal suture, and a full-thickness flap was elevated. One 4-mm-in-diameter circle critical-sized calvarial defect was surgically created in parietal bone on each side of the midsagittal suture with a 4-mm outer diameter trephine bur under sterile saline irrigation. Attention was given to avoid damaging the sagittal suture, and not to injure the dura mater beneath the bone.

Absorbable collagen membrane was cut by tissue punch into circle scaffolds with 4 mm in diameter. BMP, VEGF, PDGF, and SDF-1 were formulated into scaffolds, lyophilized, and stored at a −80°C freezer until use. They were divided into the following eight groups:

A: Scaffold only (non-crosslinking collagen membrane).

B: 1 µg SDF-1.

C: 1 µg BMP.

D: 0.5 µg VEGF.

E: 0.5 µg PDGF.

F: 1 µg SDF-1 +1 µg BMP.

G: 1 µg SDF-1 +0.5 µg PDGF.

H: 1 µg SDF-1 +0.5 µg VEGF.

The collagen membrane/chemokine/growth factor complexes were implanted into calvarial defects, and incisions were sutured. There were 4 samples for each group at each time point. Each of these four samples was implanted in a different mouse. A total of 32 mice were used. Two and four weeks after surgery, mice were sacrificed with an overdose of CO_2_. The harvested calvariae were fixed using 10% neutral buffered formalin for 2 days, and then scanned by microCT. Subsequently, all the biopsies were prepared for histology.

### MicroCT and Histology

Specimens were placed in a 34 mm diameter tube and scanned over the entire width of the specimens using a microCT system (µCT100 Scanco Medical, Bassersdorf, Switzerland). Scan settings were: voxel size 18 µm, 70 kVp, 114 cA, 0.5 mm AL filter, and integration time 500 ms.

Three dimension images were constructed with MicroView program (GE Healthcare, Waukesha, WI). Region of interest (ROI) was selected to cover whole body of the subcutaneous ectopic specimens, and a cylinder ROI with 4 mm in diameter and 2 mm in height was chosen for calvarial critical-sized defects. Tissue mineral content at ROI was measured.

After microCT analysis, the specimens were decalcified in 10% EDTA for 2 weeks, embedded in paraffin, and cut into 5 µm sections. These sections were stained routinely with hematoxylin and eosin.

### Statistical analysis

Statistical analysis was performed using Prism 4 (GraphPad Software, La Jolla, CA). All results are expressed as means ± SD. Statistical significance among multiple groups was evaluated by one-way ANOVA analysis followed by Tukey's multiple comparisons. Significance was set at p<0.05.

## Results

### Effects of SDF-1 on BMP-7-induced subcutaneous ectopic bone formation

**Figure 1:**
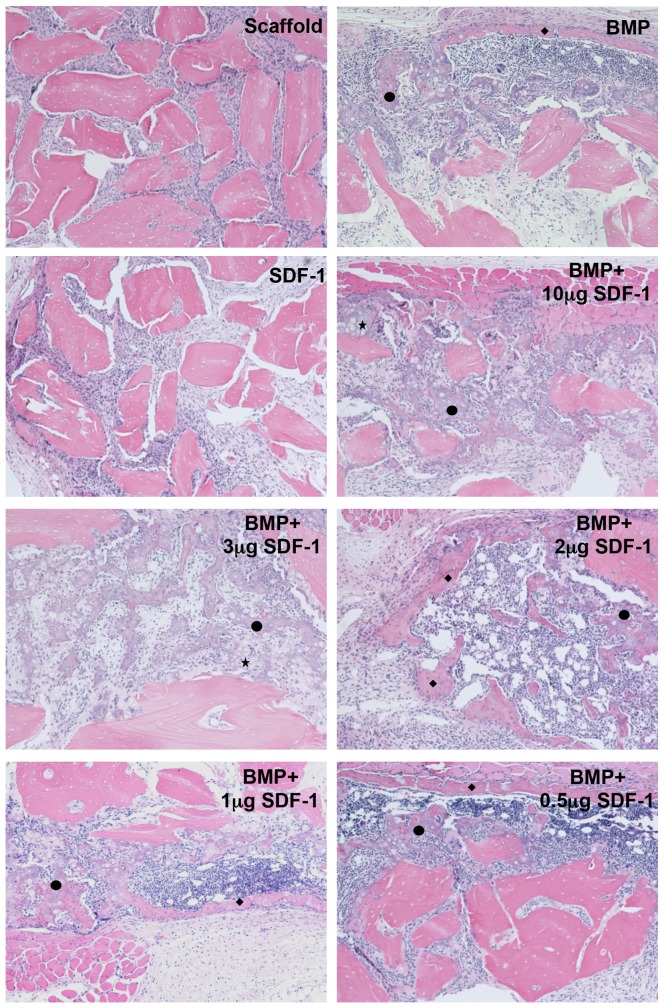
Histology of 4-week specimens of subcutaneous ectopic bone formation. Cartilage and bone formation were found at the groups with BMP, while no cartilage and bone formation was seen in scaffold only group and SDF-1 only group. More intermediate type of cartilage-bone transition was observed remaining at BMP groups containing high doses of SDF-1 (10 µg and 3 µg), while more mature bone and bone marrow-like tissues were found at BMP groups with lower doses of SDF-1 (0.5 to 2 µg). Hematoxylin and eosin staining. Magnification is 10×. * indicates cartilage, **♦** indicates bone, and • indicates intermediate type of cartilage-bone transition.

Histologically, there were no bone formation at scaffold only and SDF-1 only groups at both 2 and 4 weeks, while cartilage and bone formations on the surfaces of scaffolds were seen in all the groups containing BMP. Although cartilage still existed at 4 weeks, more intermediate type of cartilage-bone transition was observed remaining at BMP groups containing high doses of SDF-1(10 µg and 3 µg). Interestingly, more mature bone and bone marrow-like tissues were found at BMP groups with lower doses of SDF-1(0.5 to 2 µg) as is shown in Figure 1. Histology of 2- and 4-week specimens is shown in Figure S1.

**Figure 2:**
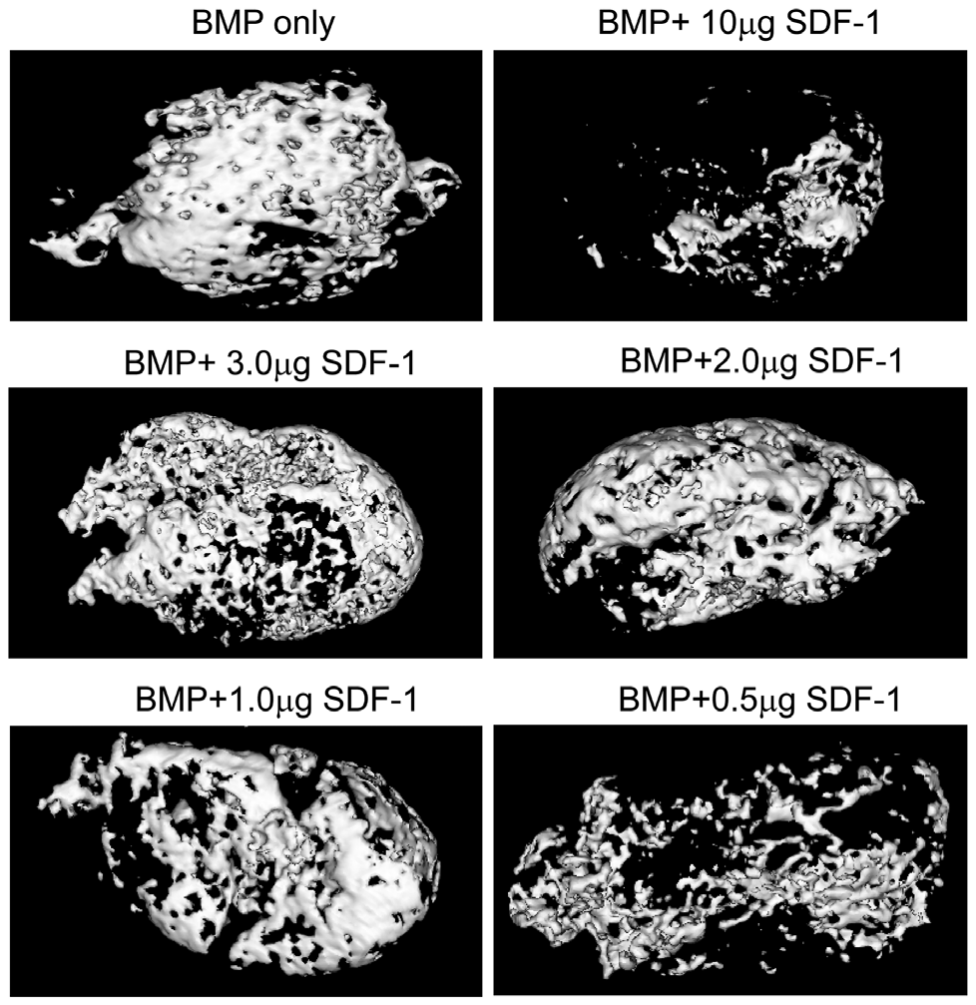
MicroCT images of SDF-1 effects on BMP-induced subcutaneous ectopic bone formation at 2 weeks. Minimal bone formation was seen at BMP group with highest dose SDF-1of 10 µg. However, other BMP groups had much more bone formation. SDF-1 only and scaffold only groups formed no bone (data not shown). MicroCT images of 4-week specimens are not shown either because there were no differences in bone formation among all BMP groups.

MicroCT images also showed that all the groups containing BMP had bone formation. Bone formation was found not only on the superficial regions of the scaffolds but also internally. Less bone formation was induced by BMP with 10 µg SDF-1 (Fig. 2). No bone formation could be detected at scaffold alone and SDF-1 groups (data not shown).

**Figure 3:**
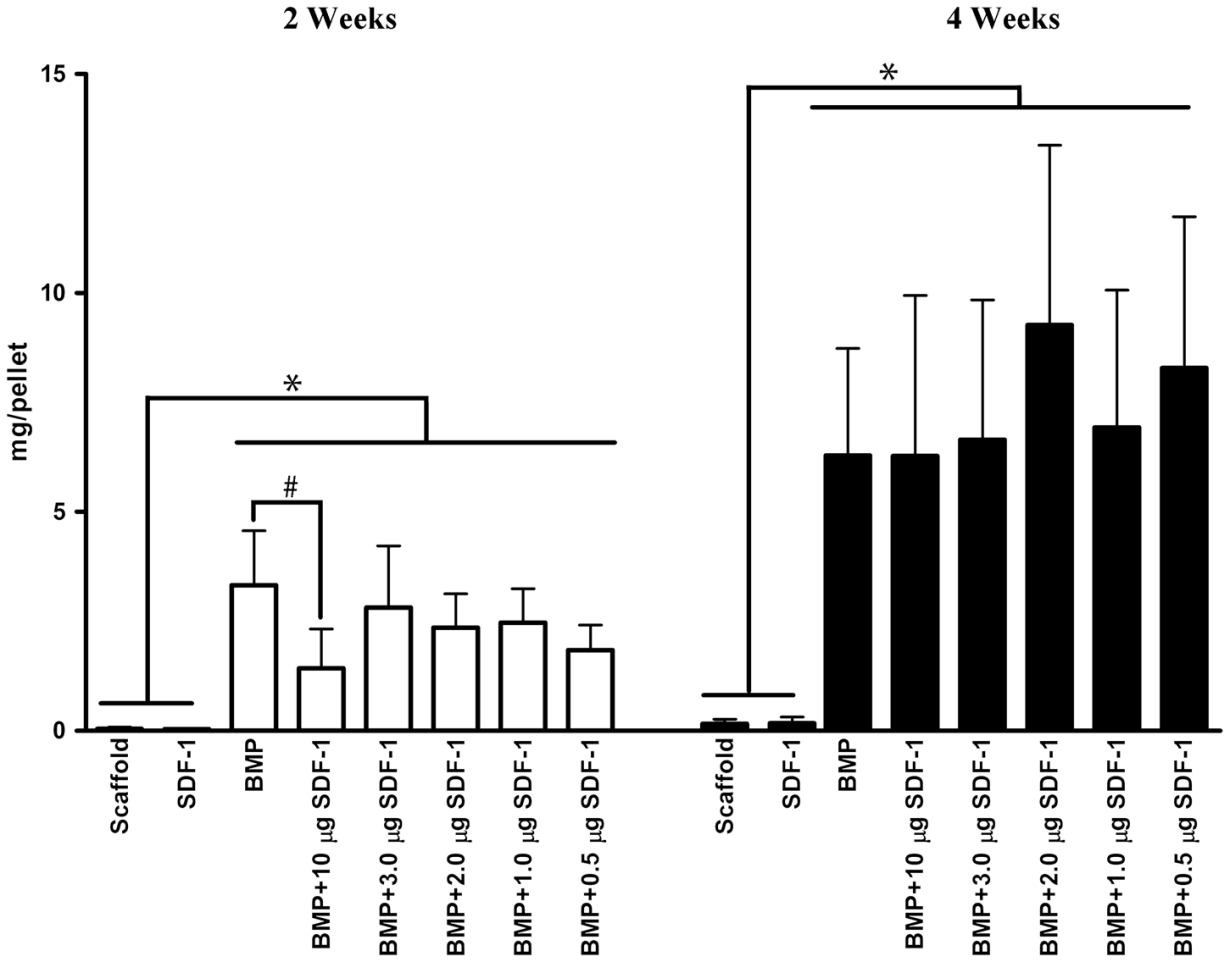
Bone mineral content of the 2- and 4-week specimens of subcutaneous ectopic bone formation. Bone mineral content analysis using microCT revealed that bone mineral content in the groups containing BMP was higher than those in the scaffold only and SDF-1 only groups at both at 2 and 4 weeks. Bone mineral content at BMP/10 µg SDF-1 delivered sites was statistically significantly lower at 2 weeks than that in BMP only group, while there were no differences among 2-week other BMP groups. At 4 weeks, no differences in bone mineral content were found among BMP groups. * represents p<0.01, # represents p<0.05. White bars represent 2-week specimens, and black bars 4-week specimens.

Consistent with the microCT imaging results, bone mineral content analysis using microCT revealed that bone mineral contents in the groups containing BMP were higher than those in scaffold only and SDF-1 only groups at both at 2 and 4 weeks. Bone mineral content in each group with BMP at 2 weeks was lower than that at each corresponding group at 4 weeks, which suggested that bone mineral content at BMP groups was increased over time (Fig. 3). Notably, bone mineral content with BMP/10 µg SDF-1 group was statistically significantly lower at 2 weeks than that in BMP only group, while there were no differences among 2-week other BMP groups, suggesting that high dose SDF-1 inhibits BMP-induced bone formation (Fig. 3). Furthermore, no statistical difference in bone mineral content was observed among BMP groups with different doses of SDF-1 except 10 µg SDF-1. This result revealed that SDF-1 has no effects on BMP-induced ectopic bone formation. By 4 weeks, there were no differences in bone mineral content among BMP groups. Thus, only 2-week microCT images are presented here.

### Effects of SDF-1 on wound healing at calvarial critical-sized defects

**Figure 4:**
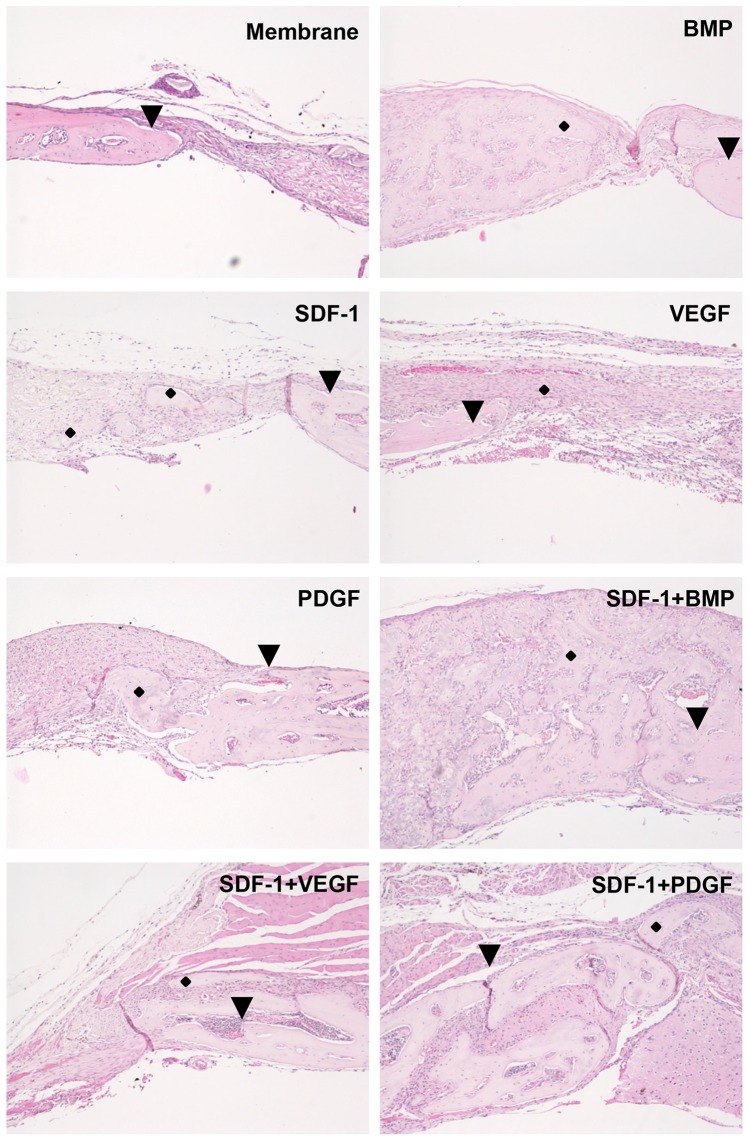
Histology of 4-week specimens of calvarial critical-sized defects. There was minimal bone formation stemming from the defect margins in the scaffold only group at 4-1, VEGF and PDGF. Hematoxylin and eosin staining. ♦ indicates bone. ▾ indicates the defect border. Magnification is 10×.

Histologically, no obvious bone formation was found in membrane only group at 2 weeks, while there was minimal bone formation stemmed from the defect margins at 4 weeks. Maximal, newly formed cartilage and bone were seen at groups containing BMP at both 2 and 4 weeks. The cartilage and bone formed not only from the defect margins but also within the collagen membrane. Noticeable bone formation was also at the defect margins in other groups containing SDF-1, VEGF and PDGF (Fig. 4). In addition, there were marcrophage-like cells seen around degrading collagen membrane fragments in all the groups. Histology of 2- and 4-week specimens is shown in Figure S2.

**Figure 5:**
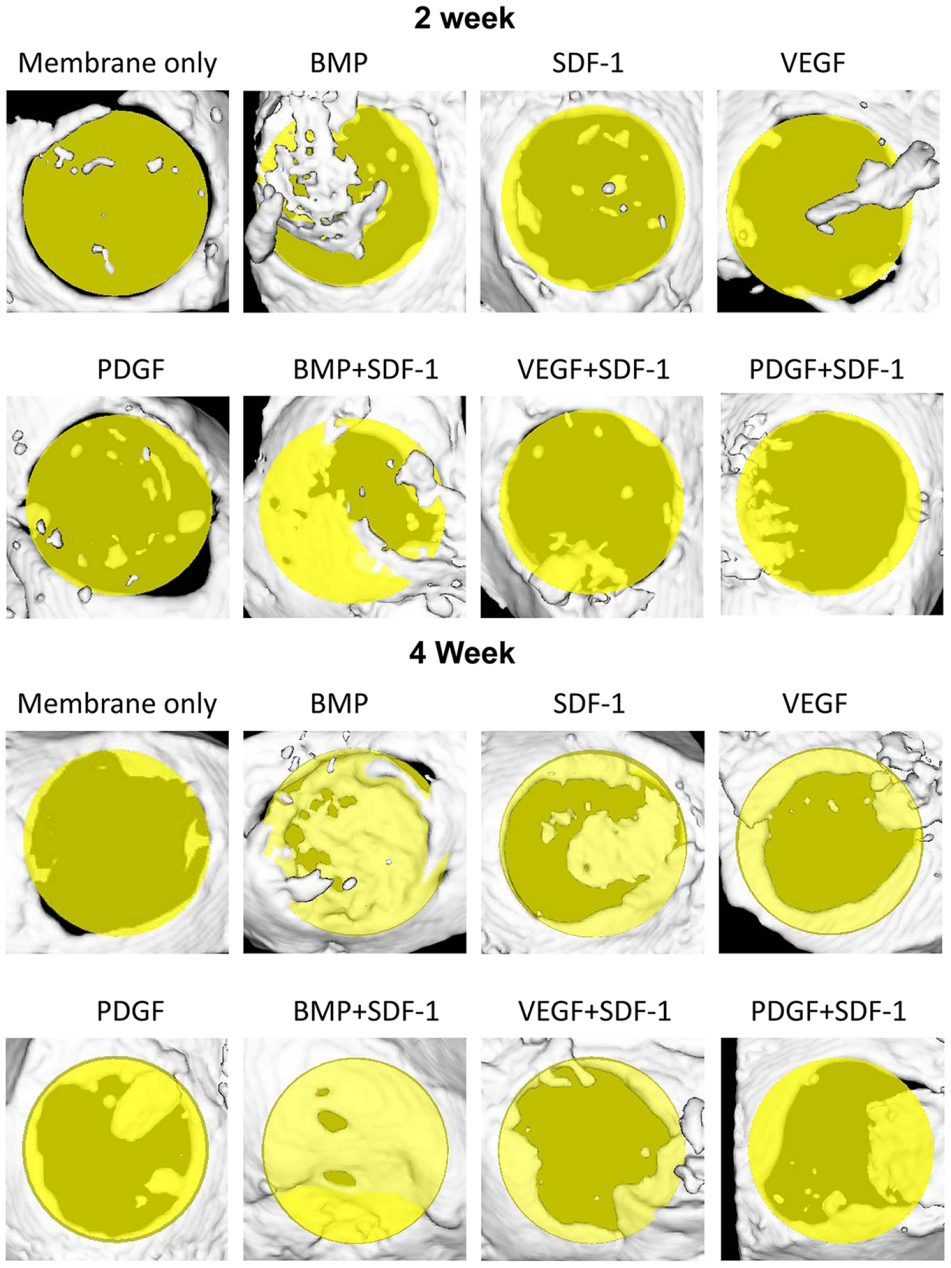
MicroCT images of the specimens of calvarial critical-sized defects. The\yellow circle plates represent original calvarial critical-sized defects. The white area above the yellow circle plate and light yellow area in the yellow plate represent newly formed bone. The membrane only group had minimal bone formation at both 2 and 4 weeks, the other groups had more obvious bone formation in the defects. Notably, groups containing BMP had nearly complete healing of defects.

Consistent with the histological findings, microCT images revealed minimal osteogenesis in the membrane only group at 2 and 4 weeks, while maximal bone formation was found in the BMP only and the BMP+SDF-1 groups at 2 and 4 weeks. In particular, at 4 weeks, the critical-sized defects were almost completely healed with new formed bone with BMP delivery (Fig. 5). Furthermore, obvious newly formed bone from defect margin was seen in groups containing SDF-1, VEGF and PDGF with greater evidence of osteogenesis at 4 weeks.

**Figure 6:**
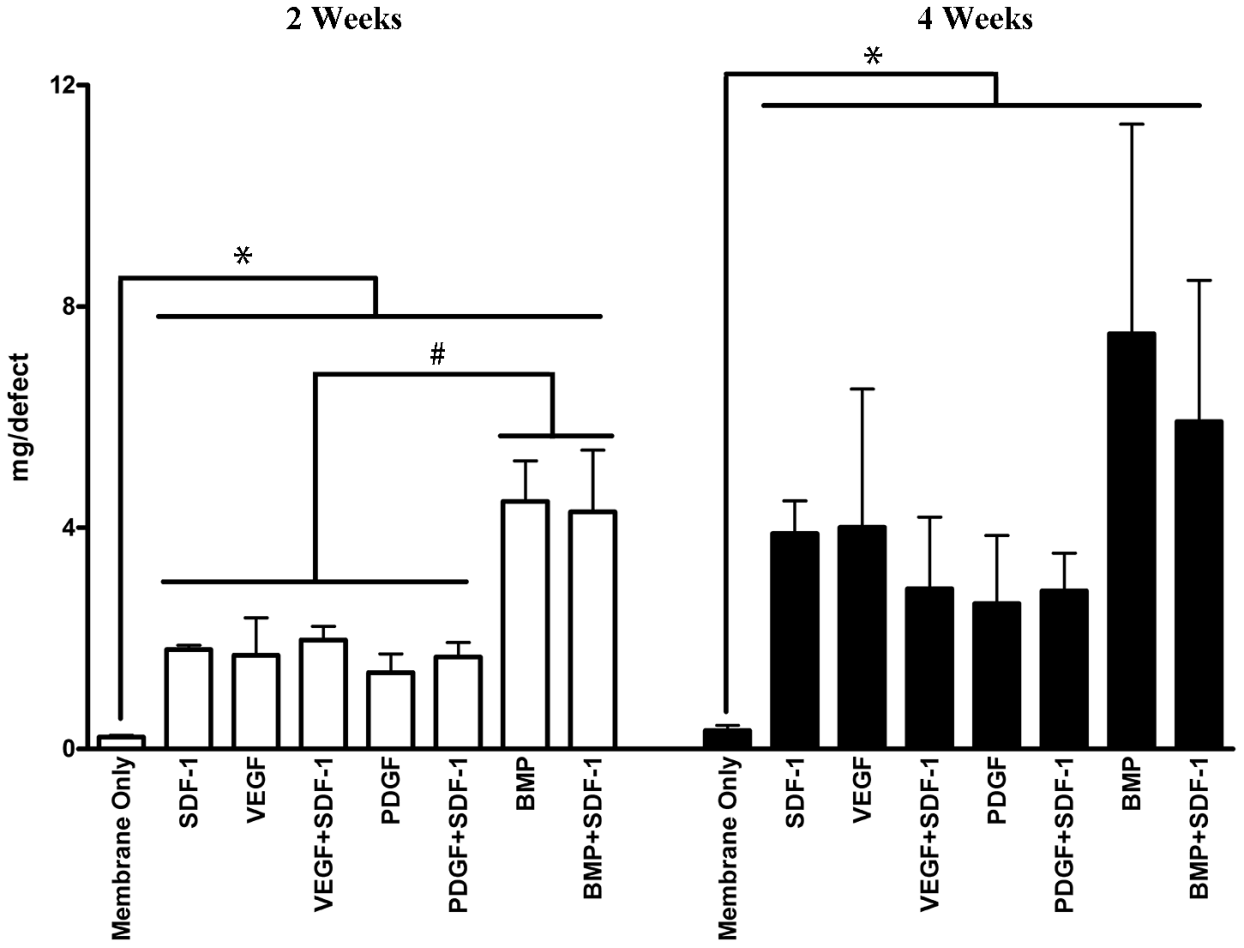
Bone mineral content of the 2- and 4-week specimens of calvarial critical-sized defects. At 2-1, VEGF, and PDGF. At 4 weeks, there were no differences in bone mineral content among the groups containing SDF-1, BMP, PDGF, and VEGF, while the membrane only group had the least amount of bone mineral content. * represents p<0.01, # represents p<0.05. White bars represent 2-week specimens, and black bars 4-week specimens.

MicroCT analysis demonstrated that defect bone mineral content in the membrane only group was statistically significantly lower than that in other groups at both 2 and 4 weeks. There was no statistical difference in defect bone mineral content among SDF-1, VEGF and PDGF groups, which indicated that SDF-1 alone has the same ability to enhance *in situ* bone healing as VEGF and PDGF. Furthermore, no statistical difference in bone mineral content was seen in the groups of VEGF+SDF-1, PDGF+SDF-1, and BMP+SDF-1 at both 2 and 4 weeks, compared to VEGF, PDGF, and BMP, respectively (Fig. 6).

## Discussion

SDF-1, as a chemoattractant to host stem cells, has been widely explored in heart, brain, kidney, bone and tooth [15], [35]–[37]. In this study, we investigated SDF-1 effects on bone formation both ectopically and in critical-sized bone defects. The results demonstrated that SDF-1 has no influence on BMP-induced ectopic bone formation, while SDF-1 does improve bone healing at calvarial critical-sized defects. We utilized two different inactive carrier delivery systems (DIBM and collagen) to evaluate ectopic and orthotopic bone formation. Both of these carriers are very comparable matrices devoid of growth factor activity as demonstrated in multiple studies. [30], [32], [34]. DIBM has been used conventionally as a BMP carrier in the ectopic model, while collagen scaffolds have been more traditionally used in the cranial defect model. The main differences that need to be highlighted here are that the ectopic model has no bone cell precursors present in the wound site, while the cranial defect has bone cell progenitors at the osteotomy margins. As such, the two different models allow for examining most closely the role of SDF-1 in the different defect model systems for *de novo* bone formation (ectopic) versus the formation of bone from an intramembranous bony defect (cranial).

Mesenchymal stem cells participating in tissue wound healing are divided into two sources: local mesenchymal stem cells and blood circulating mesenchymal stem cells. Local mesenchymal stem cells have been shown to exist in various tissues such as skin, brain, adipose and bone marrow, and also isolated successfully from these tissues [38]–[40]. In addition, circulating mesenchymal stem cells have been proven to be involved in ectopic bone formation, heart repair and brain healing. The questions here are which source of mesenchymal stem dominates healing process and how much stem cells from both sources contribute to it. Kumagai et al [41] demonstrated using a parabiotic mouse model that blood circulating stem cells account for about 10% of the total cells in mouse transverse fibular fracture callus region. About 83% of these cells occupied approximate 6% of total alkaline phosphatase positive cells, which indicates that blood circulating stem cells contribute just 6% of the total bone fracture healing. However, Otsuru et al [42] revealed that in lethally irradiated mice in which most host stem cells were killed, 40% of osteocalcin-positive cells participating in BMP-induced subcutaneous ectopic bone formation came from non-host cells in bone marrow transplantation. These results suggest that local stem cells play a major role in the healing process of a wound whose damage is within host physiological repair potential, whereas blood circulating mesenchymal stem cells contribute more to the wound healing when it is beyond host repair capacity. Because local stem cells seem to predominately control BMP-induced subcutaneous ectopic bone formation, the effects of circulating stem cells recruited by SDF-1 on bone formation become negligible. Thus, SDF-1 displayed minimal effects on BMP-induced ectopic bone formation in our present study.

In order to investigate SDF-1 effects on healing of a bony wound beyond host self-repair capacity, a murine calvarial critical-sized defect model was adopted. Our results showed that like PDGF and VEGF, SDF-1 alone could enhance critical-sized bony wound healing. Our results concur with the recent findings of Jansen and co-workers who demonstrated that SDF-1 loaded PCL/gelatin membranes yielded a 6-fold increase in the amount of bone formation compared to membranes alone at 8 weeks using the rat calvarial critical-sized defect model [43]. Much evidence demonstrates that SDF-1 plays important roles in recruitment of blood circulating stem cells into heart, brain, skin, and bone [15], [35], [39], [44]. Therefore, the ameliorative effects of SDF-1 on large critical-sized bony wound healing may result from the increase of SDF-1-chemoattracted circulating stem cells in the wound region. Secondly, SDF-1 plays crucial roles in blood vessel formation. SDF-1 knockout mice demonstrated vascularization defect in gastrointestinal tract [8]. SDF-1 also has chemotactic effect on endothelial progenitor cells [45]. Enhancement of bone formation in critical-sized defect area in this study may be associated with local angiogenesis enhancement by SDF-1. Thirdly, SDF-1 may have direct effects on osteoblast differentiation and activities, and regulate bone formation. SDF-1 receptor CXCR4 has been found in the membranes of preosteoblasts and osteoblasts. Hosogane et al. have shown that SDF-1 increased BMP-induced ALP activities and osteocalcin expression in human and mouse bone marrow stromal cells, and bone nodule mineralization was impaired when SDF-1 signaling was disrupted [46]. SDF-1 also improves ALP activities and mineralized nodule formation in human dental pulp cells [47]. These results indicated that SDF-1 enhances osteoblast differentiation. Thus, SDF-1 can accelerate calvarial critical-sized defect healing. It is necessary to further investigate which mechanism of SDF-1 dominates osteogenesis in different situations in the future.

Notably, our results found that there were no synergetic effects between SDF-1 and VEGF, and PDGF respectively on large bone defect repair beyond our expectations. It remain unclear how much blood vessel formation is necessary for SDF-1 to effectively recruit circulating stem cells. Because SDF-1 itself can enhance angiogenesis, blood vessel formation initiated by SDF-1 itself may be enough to recruit circulating stem cells temporally and spatially, though VEGF and PDGF also improve angiogenesis.

In summary, our study demonstrated that SDF-1 alone could enhance bone formation in calvarial critical-sized defect and has minimal effects on BMP-induced ectopic bone formation, which suggests that like BMP, PDGF and VEGF, SDF-1 could improve large bony defect healing beyond physiological self-repair capacity by its biological functions of circulating stem cell recruitment and enhancing local angiogenesis. In addition, the mechanisms of interactions among SDF-1, BMP, VEGF, and PDGF in bone tissue engineering are necessary for further investigation, which may lead to new effective and efficient way to treat large bone defects clinically.

## Supporting Information

Figure S1 **Histology of the specimens of subcutaneous ectopic bone formation.** Left panel is histology of 2-week specimens, and right panel is histology of 4-week specimens. Hematoxylin and eosin staining. Magnification is 10×. * indicates cartilage, **♦** indicates bone, and • indicates intermediate type of cartilage-bone transition.
(PPT)

Figure S2 **Histology of the specimens of calvarial critical-sized defects.** Left panel is histology at lower magnification 2×, and right panel is histology at higher magnification 10×. Hematoxylin and eosin staining. ♦ indicates bone. * indicates cartilage. ▾ indicates the defect border.
(PPTX)
