# Supplementary material for: SDF-1 Enhances Wound Healing of Critical-Sized Calvarial Defects beyond Self-Repair Capacity
Source: PLoS One. 2014 May 6;9(5):e97035. doi: 10.1371/journal.pone.0097035 (PMC4011888; doi:10.1371/journal.pone.0097035)
Supplement: Figure S1 — Histology of the specimens of subcutaneous ectopic bone formation. Left panel is histology of 2-week specimens, and right panel is histology of 4-week specimens. Hematoxylin and eosin staining. Magnification is 10×. * indicates cartilage, ♦ indicates bone, and • indicates intermediate type of cartilage-bone transition. (PPT) [file pone.0097035.s001.ppt]

## Slide 1
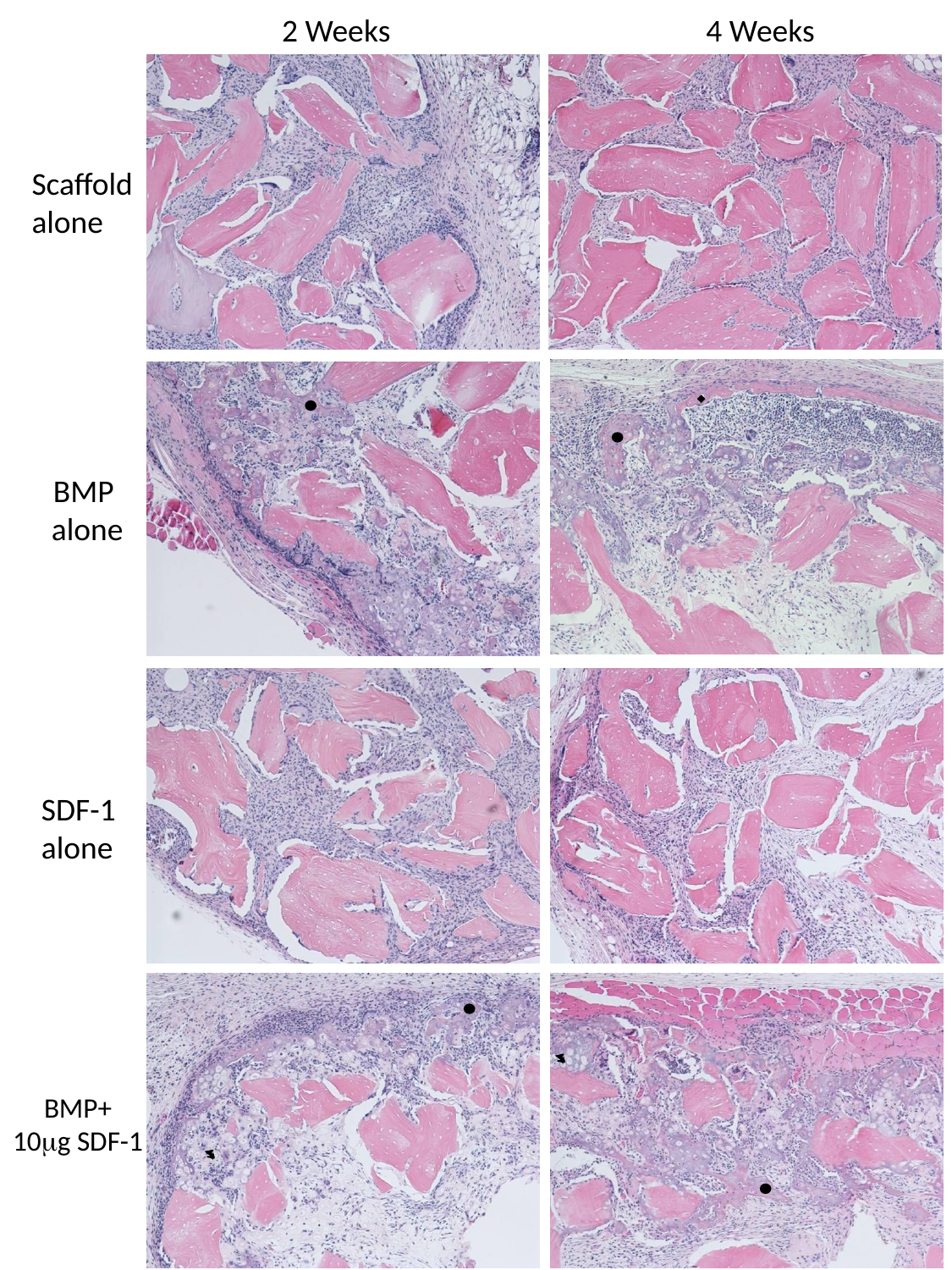

2 Weeks 4 Weeks
Scaffold
alone
BMP
alone
SDF-1
alone
BMP+
10μg SDF-1

## Slide 2
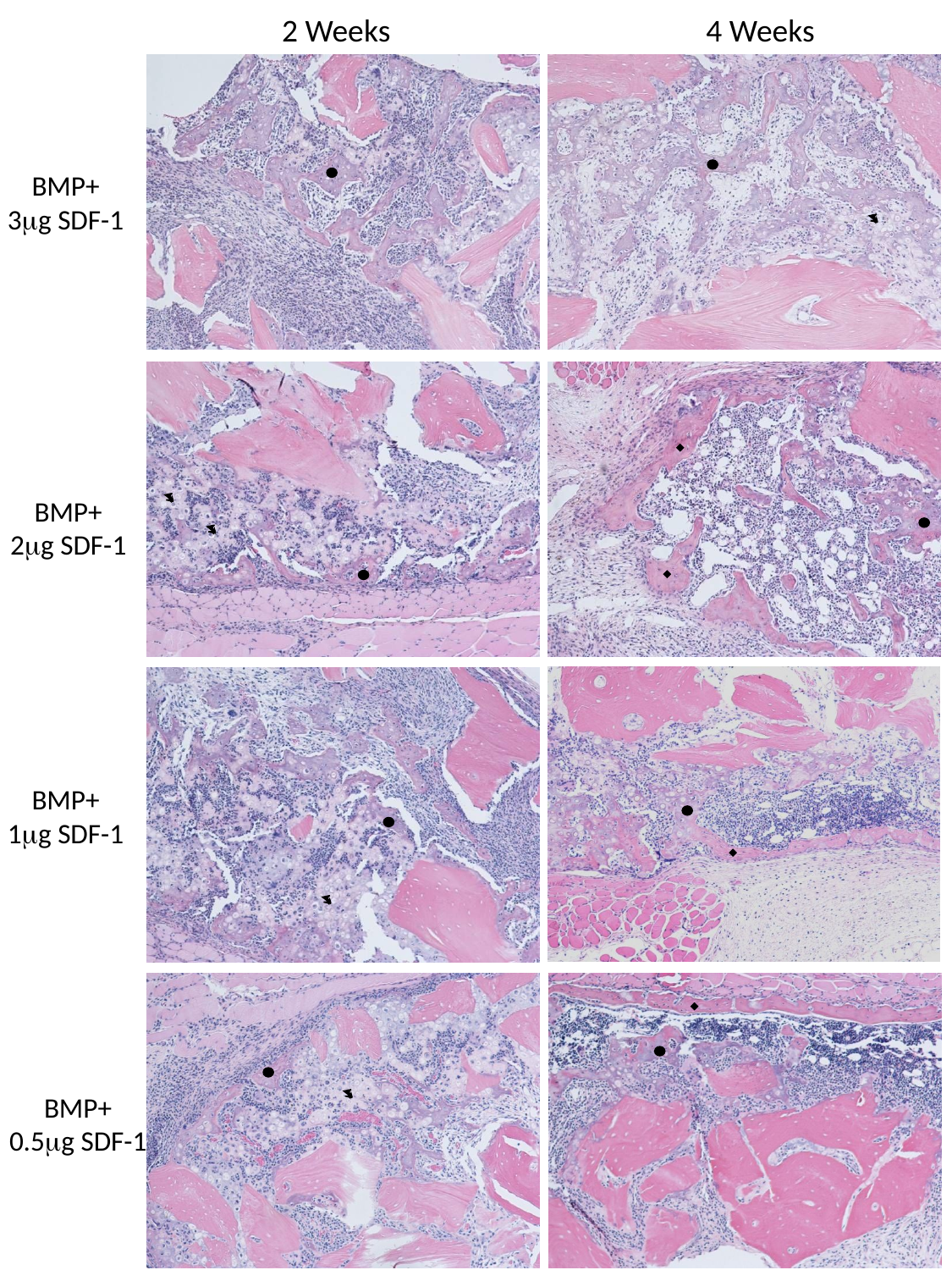

2 Weeks 4 Weeks
BMP+
3μg SDF-1
BMP+
2μg SDF-1
BMP+
1μg SDF-1
BMP+
0.5μg SDF-1
